# Assessment of the Potential Diagnostic Role of Anaplastic Lymphoma Kinase for Inflammatory Myofibroblastic Tumours: A Meta-Analysis

**DOI:** 10.1371/journal.pone.0125087

**Published:** 2015-04-24

**Authors:** Shuiqing Wu, Ran Xu, Qi Wan, Xuan Zhu, Lei Zhang, Hongyi Jiang, Xiaokun Zhao

**Affiliations:** 1 Department of Urology, The Second Xiangya Hospital of Central South University, Changsha, China; 2 Neural Medical Center of the First Hospital in Changsha City, Changsha, China; University of York, UNITED KINGDOM

## Abstract

**Objective:**

To assess the value of anaplastic lymphoma kinase for the diagnosis of inflammatory myofibroblastic tumours using a comprehensive meta-analysis.

**Methods:**

We searched the related literature using electronic databases and manual searches. Approximately 454 cases from several countries were included in this analysis. The quality of studies included was assessed by QUADAS (quality assessment of studies of diagnostic accuracy). The diagnostic odds ratio (DOR), positive likelihood ratio (PLR), negative likelihood ratio (NLR), sensitivity and specificity were calculated to assess the role of anaplastic lymphoma kinase in the diagnosis of inflammatory myofibroblastic tumours. The overall test performance was summarised by an SROC (summary receiver operating characteristic curve). The heterogeneity and publication bias were analysed using Meta-regression and Deeks' test. All data were analysed by Stata 12.0 software.

**Results:**

Eight studies were included according to our inclusion criteria. The overall results for the specificity, sensitivity, PLR, NLR, DOR and area under the curve (AUC) were 0.99 (95% CI 0.82-1.00), 0.67 (95% CI 0.46-0.83), 0.67 (95% CI 0.46-0.83), 60.6 (95% CI 3.3-1112.4), 0.33 (95% CI 0.19-0.60), 181 (95% CI 9-3684) and 0.95 (95% CI 0.93-0.97), respectively, while the specificity, sensitivity, PLR, NLR, DOR and AUC for bladder IMTs were 0.99 (95% CI 0.67-1.00), 0.86 (95% CI 0.58-0.96), 95.6 (95% CI 2.0-4616.2), 0.14 (95% CI 0.04-0.50), 671 (95% CI 16-28913) and 0.99 (95% CI 0.97-0.99), respectively.

**Conclusion:**

The present meta-analysis indicated that anaplastic lymphoma kinase plays a significant role in the differential diagnosis of inflammatory myofibroblastic tumours, particularly for inflammatory myofibroblastic tumours of the urinary bladder.

## Introduction

Inflammatory myofibroblastic tumours (IMT), also referred to as inflammatory pseudotumours, plasma cell granulomas, etc., are characterised by a fascicular proliferation of myofibroblasts with admixed inflammatory cells derived from mesenchymal tissue [1]. IMTs are rare entities, and the disease incidence of these tumours remains unclear. These tumours can arise in the respiratory and genitourinary system, particularly in the lung and bladder; however, IMTs have also been reported in other sites [2–3]. IMTs are considered borderline tumours, indicating an intermediate biological potential for recurrence or metastasis [4]. However, their clinical and pathological manifestations mimic malignancy; therefore, it is difficult but important to differentiate IMTs from other soft tumours. A false diagnosis may cause great harm to patients [5]. Therefore, the discovery of a potential biomarker to improve the diagnostic accuracy of IMTs is imperative.

IMT diagnosis remains challenging due to overlapping immunohistochemical and morphological characteristics. The anaplastic lymphoma kinase (ALK) is detected in the majority of IMTs and is negative in several other soft tissue tumours. The ALK gene is located on chromosome 2p23 and belongs to the insulin receptor family of tyrosine kinases [6].

Since the discovery of 2p rearrangements in IMTs in 1999 [7], ALK has been considered a promising biomarker for improving the diagnostic accuracy of IMT, specifically during differential diagnosis. However, controversy remains concerning the diagnostic role of ALK in the diagnosis of IMTs. We performed a primary meta-analysis to assess the potential diagnostic role of anaplastic lymphoma kinase for Inflammatory Myofibroblastic Tumours.

## Materials and Methods

### Search strategy and study selection

A comprehensive literature search for suitable studies published before July 23, 2014 was conducted in the following electronic databases, Pubmed, Embase, Web of Science, Cochrane Library and 3 Chinese databases: Wan Fang, Chinese National Knowledge Infrastructure (CNKI), Chinese Biology (CBM), and a manual search in the medical library of the Central South University. Studies that investigated the diagnostic role of anaplastic lymphoma kinase in inflammatory myofibroblastic tumour (IMT) diagnosis were included in this meta-analysis. Studies had to be published as a full paper with a publish date prior to July 2014 but with no lower date limit. The search was conducted using the following keywords: “anaplastic lymphoma kinase or ALK or ALK-1”, “inflammatory myofibroblastic tumour or inflammatory pseudotumour or IMT” and “diagnostic value or diagnosis or ROC curve or sensitivity or specificity”.

### Inclusion and exclusion criteria

Studies eligible for inclusion in this meta-analysis met the following criteria: (1) studies regarding the diagnostic role of anaplastic lymphoma kinase for inflammatory myofibroblastic tumour; (2) when duplicate articles were published, only the newest or most informative single article was selected; (3) studies provided sufficient data for the construction of 2-by-2 tables, including true positive (TP), false positive (FP), true negative (TN), and false negative (FN). The exclusion criteria were as follows: (1) studies did not include raw data, such as reviews, letters, case reports, conference abstracts and editorials; (2) publications not related to the diagnostic role of anaplastic lymphoma kinase for inflammatory myofibroblastic tumour; (3) there was no control group in the study.

### Data extraction and quality assessment

Two reviewers (SQW and LZ) independently extracted the following data from all of the included articles: author, publication year, country, location, detection method, the number of patients diagnosed with IMT, the number of controls, sample size, true positive (TP), false positive (FP), true negative (TN), false negative (FN), specificity, sensitivity. The studies were assessed for methodological quality according to the QUADAS (quality assessment of diagnostic accuracy studies) criteria [8]; one of these items, the avoidance of disease progression bias, was not included because it was not relevant to this study. Each item is answered with “yes,” “no,” or “unclear” response. An answer of “no” or “unclear” indicates that the risk of bias may be high and an answer of “yes” indicates that the risk of bias is low. An additional reviewer (XKZ) assessed all discrepancies and the majority opinion was used to resolve disagreements between the reviewers.

### Statistical analysis

The standard methods recommended for diagnostic accuracy meta-analysis were used in this study [9]. We extracted the numbers of participants with TP, TN, FP, and FN results from every included study and the sensitivity, specificity, positive likelihood ratio (PLR), negative likelihood ratio (NLR), diagnostic odds ratio (DOR) and area under the summary receiver operating characteristic curve (SROC) were analysed using the bivariate meta-analysis model. The statistical analysis was performed using STATA 12.0 software. The area under the curve (AUC) of the SROC indicates the ability to distinguish IMTs from controls. An AUC close to 0.5 indicates a poor diagnostic test while an AUC close to 1.0 suggest good accuracy for the test [10]. The variations and heterogeneities of the results across studies were examined using the Cochran’s Q-statistic and Chi-square test [11]. Heterogeneity was considered significant when the I^2^ value was greater than 50% or the Q test’s p value was less than 0.1. Meta-regression or subgroup analyses were performed if heterogeneity was observed to explore the sources of heterogeneity. The publication bias was analysed using Deeks’ test [12].

## Results

### Literature outcomes

**Fig 1:**
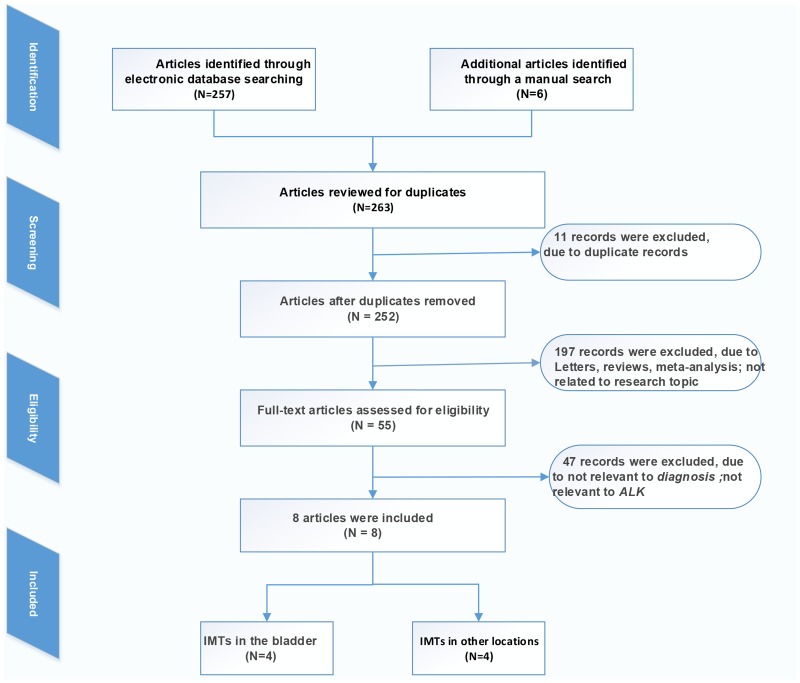
Flow diagram of the study selection process.

Our research yielded 263 primary studies after the initial independent review, comprising 257 literature sources identified through electronic database searches and 6 literature sources identified by a manual search. Fig 1 shows the flow of studies through the review process. Eleven records were initially excluded due to duplicate records, 197 records were excluded due to the source being a letter, review, meta-analysis or not related to the research topic; 47 records were excluded due to not being relevant to diagnosis or not relevant to ALK. Finally, 8 studies [13–20] fulfilled all the inclusion criteria and were considered for analysis, with 4 literature sources focused on IMTs in the bladder and the other 4 sources focused on other locations (not reported), as illustrated in Table 1.

**Table 1:** Characteristics and quality assessment of the included ALK studies.

Study ID	Country	Sites	Detection Method	IMTs(n)	Control(n)	Sensitivity	Specificity	Sample Size
Cook JR,2001	USA	NR	IHC	73	soft tissue tumours(50)	60.27%	100%	123
Cessna MH,2002	USA	NR	IHC	10	soft tissue tumours (125)	30%	92%	135
Tsuzuki T,2004	USA	Bladder	IHC	16	sarcomas(15)	75%	100%	31
Freeman A,2004	LONDON	Bladder	IHC	9	Sarcomas,NF(11)	88.89%	100%	20
Montgomery,2006	USA	Bladder	IHC	20	sarcomas(17)	100%	88.24%	37
Sukov WR,2008	USA	Bladder	IHC	21	sarcomas(31)	61.90%	100%	52
Mao R,2008	CHINA	NR	IHC	15	AF;GIST(10)	46.67%	100%	25
Qiu X,2008	CHINA	NR	IHC	22	sarcomas(9)	40.91%	100%	31

N number, NR not reported, IHC immunohistochemistry, IMTs inflammatory myofibroblastic tumours, AF abdominal fibromatosis, GIST gastrointestinal stromal tumour,NF neurofibromas

### Assessments of literature quality

**Fig 2:**
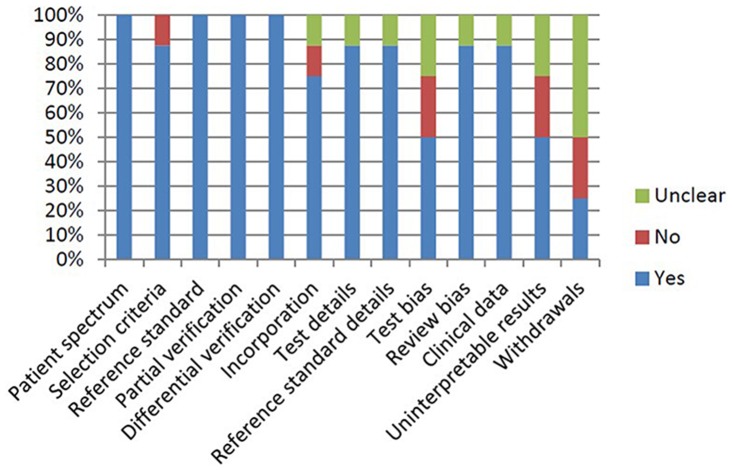
Results of quality assessment for appropriate patient spectrum studies.

The results from the assessment of the literature’s quality are shown in the bar graph in Fig 2. The records were assessed for methodological quality according to the QUADAS criteria [21]. However, one of the items, the avoidance of disease progression bias, was not included because it was not relevant to this study. There was generally good quality in the included studies; only 3 QUADAS items were not met by 70% of the studies (test bias, uninterpretable results and withdrawals).

### Diagnostic role of ALK in IMTs

**Fig 3:**
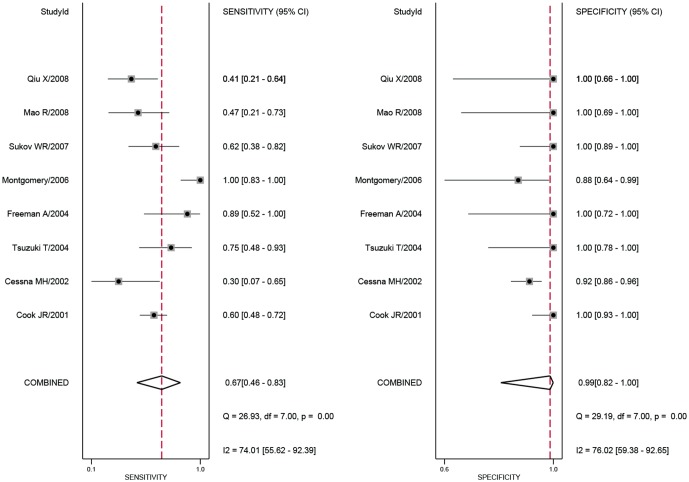
The overall forest plots of the estimates of sensitivity and specificity.

**Fig 4:**
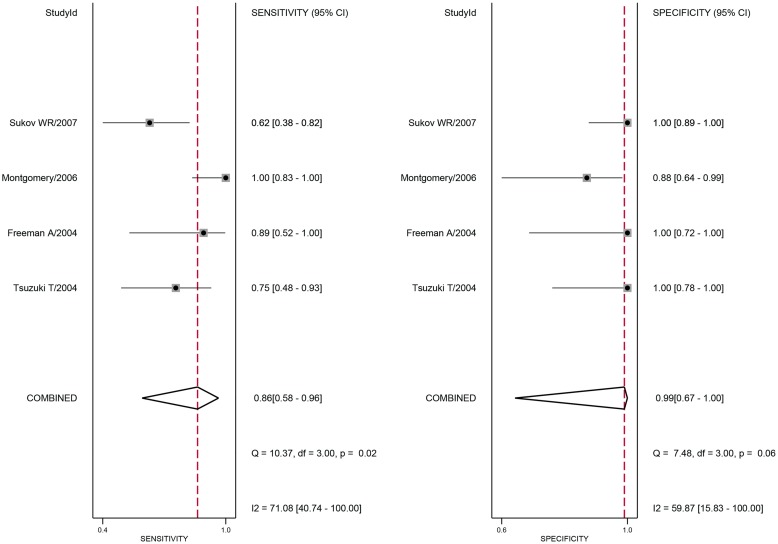
Forest plots of the estimates of sensitivity and specificity of bladder inflammatory myofibroblastic tumours.

**Fig 5:**
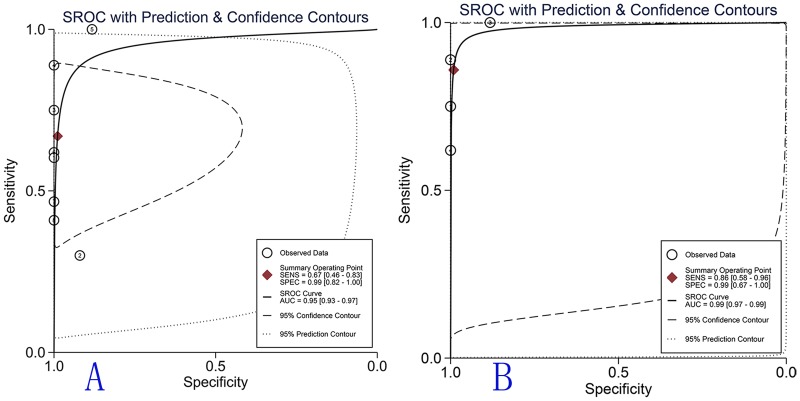
SROC curve for ALK in the diagnosis of IMTs. A. The overall SROC curve for ALK in IMT diagnosis in the 8 included studies. B. The SROC curve for ALK in bladder IMT diagnosis. The solid circles represent each study included in the meta-analysis. The size of each study is indicated by the size of the solid circle.

The pooled estimates for the diagnostic accuracy of ALK in inflammatory myofibroblastic tumours are presented by group in Table 2. The random effects model was used in this meta-analysis because significant heterogeneity was observed between studies. Fig 3 shows the forest plots for the pooled sensitivity and specificity of anaplastic lymphoma kinase for diagnosing inflammatory myofibroblastic tumours. The overall sensitivity and specificity (95% CI) were 0.67 (0.46–0.83) and 0.99 (0.82–1.00), respectively. The overall positive likelihood ratio (PLR) and negative likelihood ratio (NLR) were 60.6 (3.3–1112.4) and 0.33 (0.19–0.60), respectively, and the overall diagnostic odds ratio (DOR) was 181 (9–3684). Additionally, Fig 4 illustrates the pooled results concerning the diagnostic accuracy of bladder IMTs. The SROC curve for anaplastic lymphoma kinase is illustrated in Fig 5 and the overall area under the curve (AUC) was 0.95 (0.93–0.97) which indicates a relatively high level of diagnostic accuracy. The SROC curve for anaplastic lymphoma kinase in the diagnosis of IMTs in the bladder or other sites (NR) is illustrated in Fig 5B and S2 Fig, and the AUCs were 0.99 (95% CI 0.97–0.99) and 0.72 (95% CI 0.68–0.76), respectively. These results indicate that anaplastic lymphoma kinase assays are important tools for discriminating inflammatory myofibroblastic tumours from sarcomas and other lesions in soft tissue.

**Table 2:** Summary results for the ALK diagnostic accuracy for inflammatory myofibroblastic tumours.

Subgroup	No. of Studies	Sensitivity (95% CI)	Specificity (95% CI)	Positive LR (95% CI)	Negative LR (95% CI)	DOR (95% CI)
Bladder	4	0.86(0.58–0.96)	0.99(0.67–1.00)	95.6(2.0–4616.2)	0.14(0.04–0.50)	671(16–28913)
NR	4	0.47(0.33–0.62)	0.98(0.73–1.00)	29.9(1.3–705.4)	0.53(0.40–0.72)	56(2–1544)
Overall	8	0.67(0.46–0.83)	0.99(0.82–1.00)	60.6(3.3–1112.4)	0.33(0.19–0.60)	181(9–3684)

CI confidence interval, LR likelihood ratio, DOR diagnostic odds ratio

### Meta-regression and subgroup analyses

**Fig 6:**
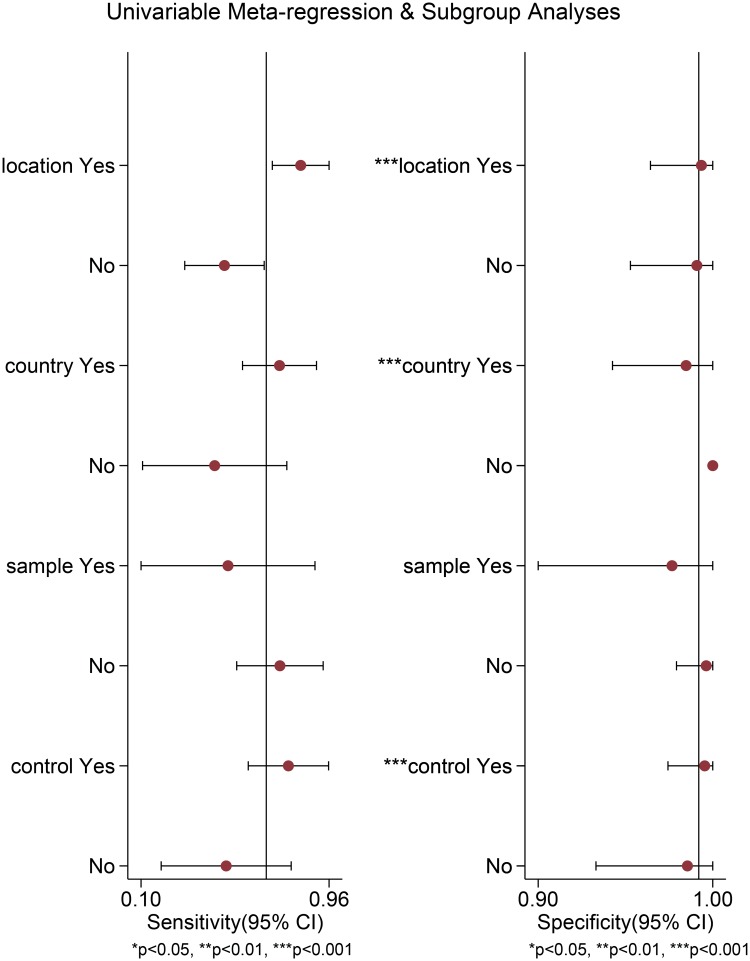
Univariate meta-regression for the sensitivity and specificity of ALK for IMT diagnosis.

Heterogeneity, which may exist across the included studies, was assessed using meta-regression and subgroup analyses. We used 4 covariates in the meta-regression and subgroup analyses: (1) Location (Lung/NR = 0, Bladder = 1), (2) country (China = 0, America/London = 1) (3) sample size (<60 = 0, ≥60 = 1), and (4) control (soft tissue lesions = 0, sarcomas = 1). Fig 6 shows the results of the meta-regression and subgroup analyses. As shown in Fig 6, the above covariates were not found to be major sources sensitivity heterogeneity. The 4 covariates may significantly contribute to specificity heterogeneity, specifically the country and location covariates. Table 2 presents the pooled results for the diagnostic accuracy of the various locations.

### Assessment of publication bias

**Fig 7:**
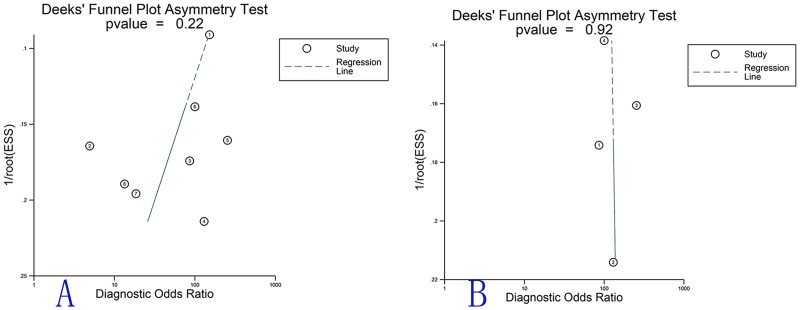
The overall Deeks' Funnel plots. A. The overall Deeks' test of the diagnostic meta-analysis of the 8 included studies; the overall p value was 0.22. B. The Deeks’ test for ALK in diagnosis of bladder IMTs; the p value was 0.92. The dotted line indicates the regression line.

The publication bias of the studies was assessed using the Deeks’ funnel plot asymmetry test. The slope coefficient of the 8 studies was associated with a p value of 0.22 (Fig 7A) and the p value of the studies concerning IMTs in the urinary bladder and other sites (NR) were 0.92 (Fig 7B) and 0.11 (S3 Fig), respectively. The above results indicate symmetrical data and no significant publication bias.

## Discussion

Inflammatory myofibroblastic tumours (IMT) are myofibroblastic proliferations in the diseased region, which are termed nodular fasciitis, plasma cell granuloma, inflammatory pseudotumour, pseudosarcoma, etc. In 1973, the first report concerning inflammatory myofibroblastic tumours (IMT) in a lung lesion was authored by Bahadori and Liebow [22]. Additionally, the first report representing IMT of the urinary bladder was described by Roth in 1980 [23]. The lung is the most-frequently reported site of this lesion, and the urinary bladder is the most common site of this lesion in the genitourinary tract. The most common symptom of IMT is painless haematuria [24]. Currently, IMT is considered a rare soft tissue tumour that has a good prognosis. It is difficult to differentiate between IMT and soft tissue tumours due to their overlapping histologic features, such as sarcomas, malignant fibrous histiocytoma, fibromatosis, etc. [25]. An IMT lesion may be easily misdiagnosed as a malignant tumour due to its hypervascularity, which could lead to severe results [5].

Anaplastic lymphoma kinase (ALK) is a protein encoded by the ALK gene in chromosome 2p23. The first report detailing ALK rearrangements was described in patients who were diagnosed with anaplastic large cell lymphoma (ALCL). Approximately 50% to 80% of ALCL cases present with ALK, particularly in young patients [26]. Clonal genetic aberrations in the short arm of chromosome 2 have been detected in almost 50% to 60% of IMTs. Chromosome 2p23, including the ALK gene, is involved in these rearrangements. ALK expression, associated with chromosome 2p23 rearrangements, has been demonstrated using specific antibodies in 20%-89% of IMTs. Additionally, clonal genetic aberrations involving the ALK gene were detected using fluorescence in situ hybridisation in 50% to 60% of IMTs [24]. The detection of anaplastic lymphoma kinase (ALK) using immunohistochemistry (IHC) or ALK gene rearrangements using FISH is important for distinguishing IMTs from other soft tissue tumours, which is fundamental for guiding clinical treatment and avoiding associated complications.

Anaplastic lymphoma kinase (ALK) is overexpressed in IMTs and ALCLs compared with normal tissue and other soft tissue tumours, particularly sarcomas. Cook JR et al. [13] analysed ALK expression in IMTs and other spindle cell processes by immunohistochemistry (IHC) using an anti-ALK antibody (ALK-1). The results showed that ALK positivity was detected in 44 of 73 (60%) IMTs, while all cases (50 cases) of other spindle cell proliferations were ALK negative (p<0.001). The data indicated that ALK positivity is highly specific to IMT diagnosis. Coffin CM et al. [27] analysed the immunohistochemical expression of ALK and other markers in a subset of IMTs with histologic atypia and/or clinical aggressiveness. The results showed that ALK positivity was detected in 33 of 59 (56%) IMTs and ALK positivity was associated with local recurrence but not distant metastasis, which indicated that ALK may be a favourable prognostic maker for local relapse. Rao RN et al. [28] reported a case of a 27-year-old female who presented with painless haematuria, burning micturition, clots in the urine, which mimicked malignant bladder tumours. ALK immunostaining was strongly present in the lesions, indicating that ALK overexpression is helpful for distinguishing IMT from malignant tumours in the urinary bladder. Takeshita H et al. [29] presented a case of a 52-year-old woman. The pathological results after transurethral resection indicated sarcomatoid carcinoma; she then underwent anterior pelvic exenteration and the building of a continent reservoir. However, the final diagnosis was corrected to an IMT due to ALK positivity and epithelial growth in the tumour cells, resulting in a good patient prognosis. These findings indicate that ALK expression is common in IMTs. ALK, as a diagnostic marker, may have some advantages for differential diagnosis [30]. ALK positivity may be able to confirm the diagnosis of IMT in specimens, specifically for suspicious diagnoses.

In this meta-analysis, 8 studies were included according to the inclusion criteria. The quality of the studies was assessed using the QUADAS criteria; the quality of the included studies was generally good with 11 of 13 QUADAS items surpassing 70% in the included studies. The heterogeneity among the selected studies was analysed using the I-squared and Chi-square (Q and p value) values. The overall inconsistency (I-squared) was 60%, while the Q and p values were 5.0 and 0.041, respectively. The pooled specificity and sensitivity values were 0.99 (95% CI 0.82–1.00) and 0.67 (95% CI 0.46–0.83), respectively. The accuracy of the ALK test to distinguish IMTs from sarcomas and other soft tissue tumours was determined by the area under the curve (AUC) of receiver operating characteristic (ROC); the AUC value was 0.95 (95% CI 0.93–0.97). An additional indicator of the diagnostic accuracy of this test, ranging from 0 to infinity, was the diagnostic odds ratio (DOR). The diagnostic odds ratio represents the frequency of positive test results among patients with or without the condition of interest, and higher DOR values always indicate better diagnostic accuracy [31]. The DOR of this meta-analysis was 181 (95% CI 9–3684),and the overall heterogenicity of specificity and sensitivity may contribute to the wide range of the 95% CI for DOR. To sum up, the above results, possessing a high level of overall accuracy, indicate that ALK is a good biomarker for the differential diagnosis of IMTs.

According to the literature, the bladder is a predilection site of IMT; therefore, a subgroup analysis regarding the different tumour sites was conducted among the studies. In the present meta-analysis, the pooled sensitivity and specificity of bladder IMTs were 0.86 (95% CI 0.58–0.96) and 0.99 (95% CI 0.67–1.00), respectively, and the AUC and DOR were 0.99 (95% CI 0.97–0.99) and 671 (95% CI 16, 28913), respectively. However, the results for sensitivity, specificity, AUC and DOR were 0.47 (95% CI 0.33–0.62), 0.98 (95% CI 0.73–1.00), 0.72 (95% CI 0.68–0.76) and 56 (95% CI 2–1544), respectively, for the diagnostic accuracy of IMTs in other sites (NR). The above data suggest that ALK has a better diagnostic performance in bladder IMTs compared with tumours in other sites.

Based on the above data, heterogeneity was detected among the included studies. The meta-regression and subgroup analyses were performed to analyse the potential sources of heterogeneity. The p value of the overall Deeks' test was 0.22 and the p value of the studies concerning IMTs in the urinary bladder and other sites (NR) were 0.92 (Fig 7B) and 0.11(S3 Fig), which indicated that the publication bias was not statistically significant. The results of the meta-regression and subgroup analyses suggested that the heterogeneity was not significant for sensitivity; however, the heterogeneity for the specificity of various sites, countries and controls was still evident. Simultaneously, we address several limitations in the present meta-analysis. First, there were a total of 454 cases analysed in our study because IMT is a rare clinical entity and the related reports are limited. Therefore, additional related studies are required to further confirm the above findings. Moreover, the IMT sites were not definitely differentiated in a few of the included studies and the NR subgroup sites may involve bladder IMTs. Finally, the control groups were generally cases that mimicked the pathological characteristics of IMTs or their clinical manifestation, which indicated the significant role of ALK in differential diagnosis of IMT. Additionally, the soft tissue tumour control group may include some sarcomas. Generally, the above factors may affect the diagnostic accuracy of ALK for IMTs.

In conclusion, IMT is a rare entity and it may be easily misdiagnosed as a malignant tumour, which may cause severe results for patients. The present meta-analysis systematically indicated that the ALK protein plays a significant role in the differential diagnosis of IMT, particularly for bladder IMTs. However, further investigations and additional improvements are required to validate the diagnostic accuracy of ALK. To improve the diagnostic performance of ALK in IMTs, the ALK test should be combined with other biomarkers as well as conventional and clinical parameters.

## Supporting Information

S1 Fig Forest plots of the sensitivity and specificity estimates for IMTs in other sites. (TIF)

S2 Fig The SROC curve for ALK in IMT diagnosis in other sites. (TIF)

S3 Fig The Deeks’ test for ALK in IMT diagnosis in other sites. (TIF)

S4 Fig The primary date extracted from the included studies. (TIF)

S1 File PRISMA Checklist. (DOC)

S2 File A list of full-text excluded articles with the reasons for exclusion. (XLS)
