# Supplementary figures and images for: Assessment of the Potential Diagnostic Role of Anaplastic Lymphoma Kinase for Inflammatory Myofibroblastic Tumours: A Meta-Analysis
Source: PLoS One. 2015 Apr 24;10(4):e0125087. doi: 10.1371/journal.pone.0125087 (PMC4409171; doi:10.1371/journal.pone.0125087)

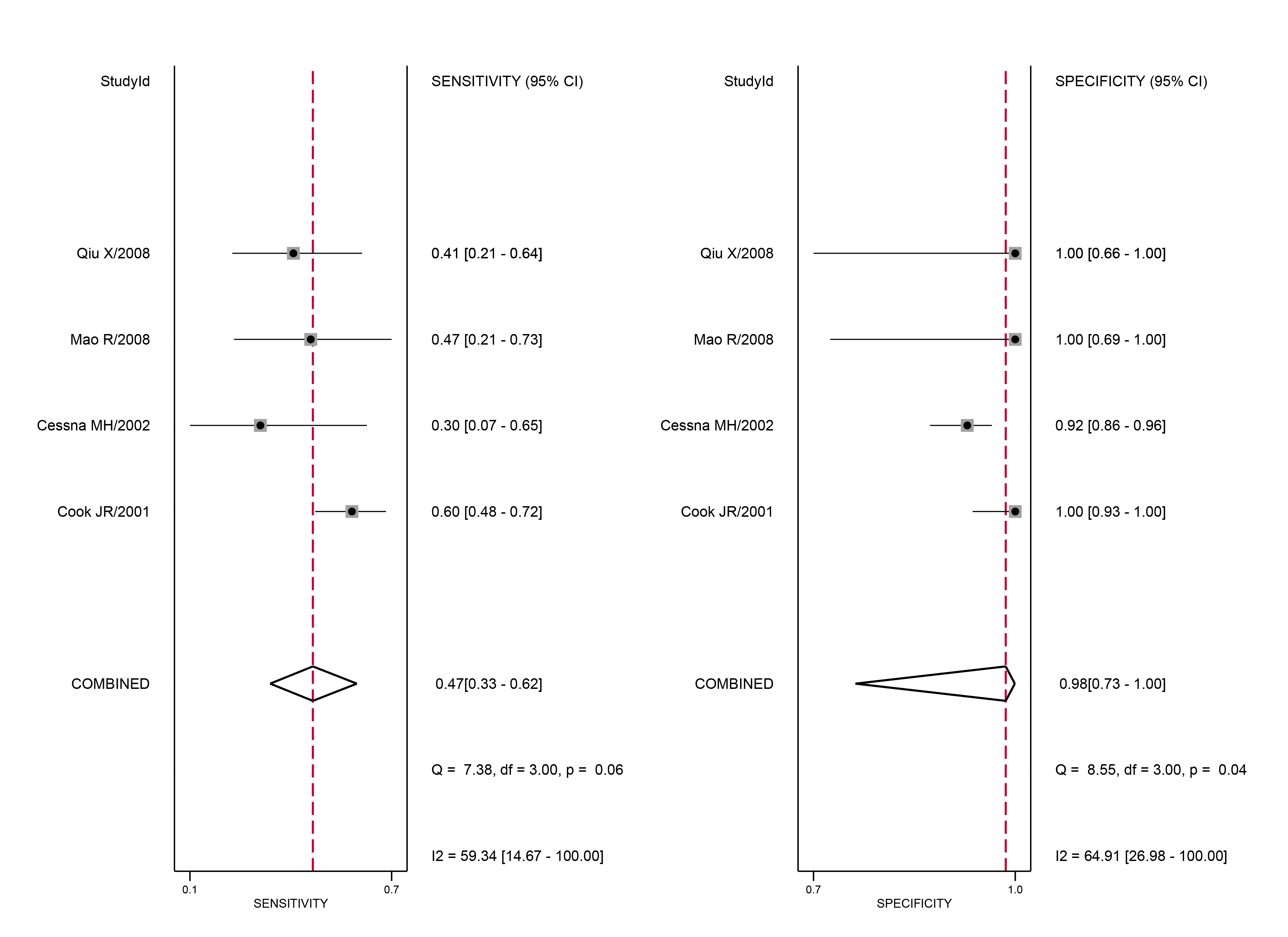

Supplement: S1 Fig — (TIF) [file pone.0125087.s001.tif]

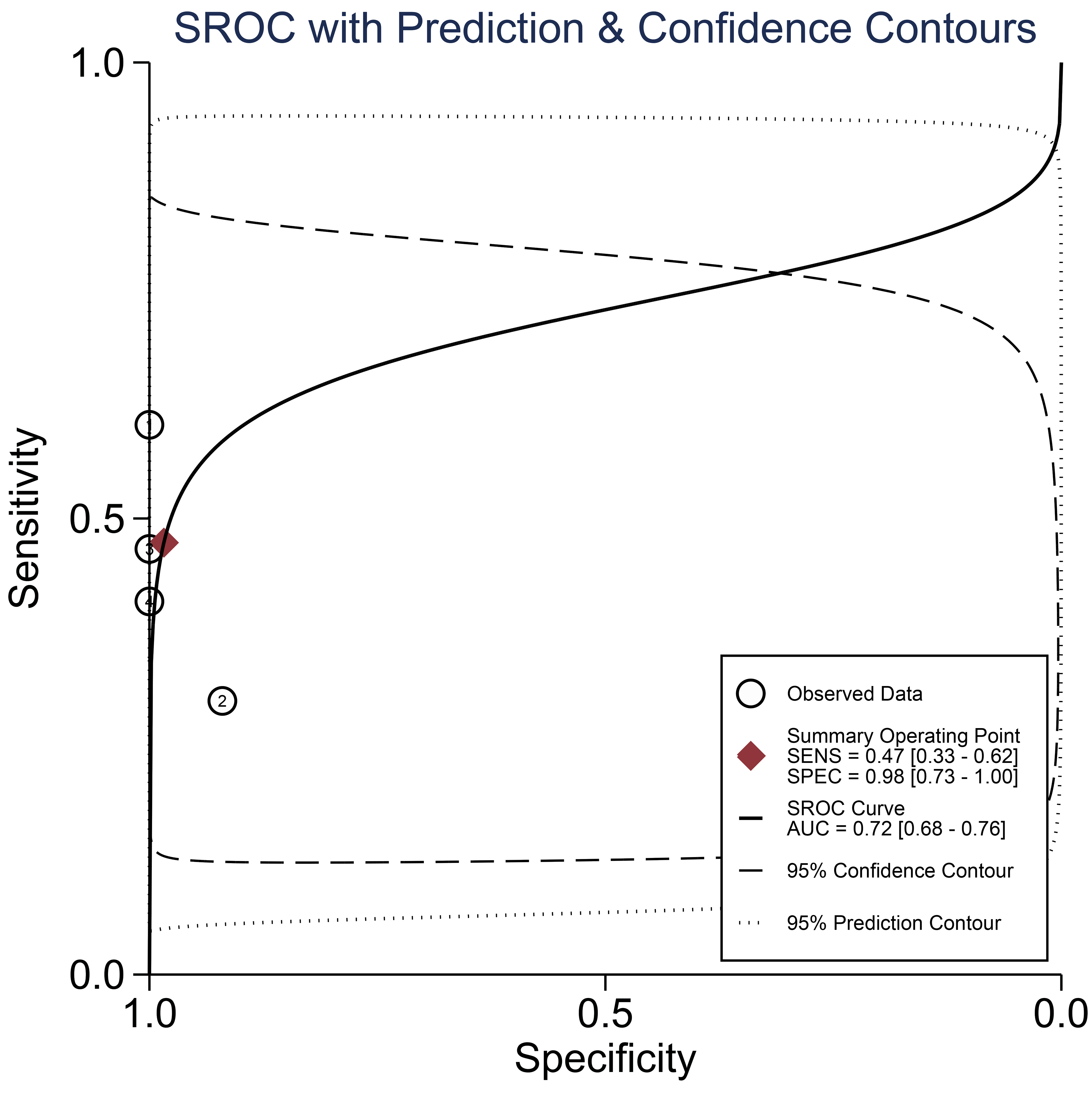

Supplement: S2 Fig — (TIF) [file pone.0125087.s002.tif]

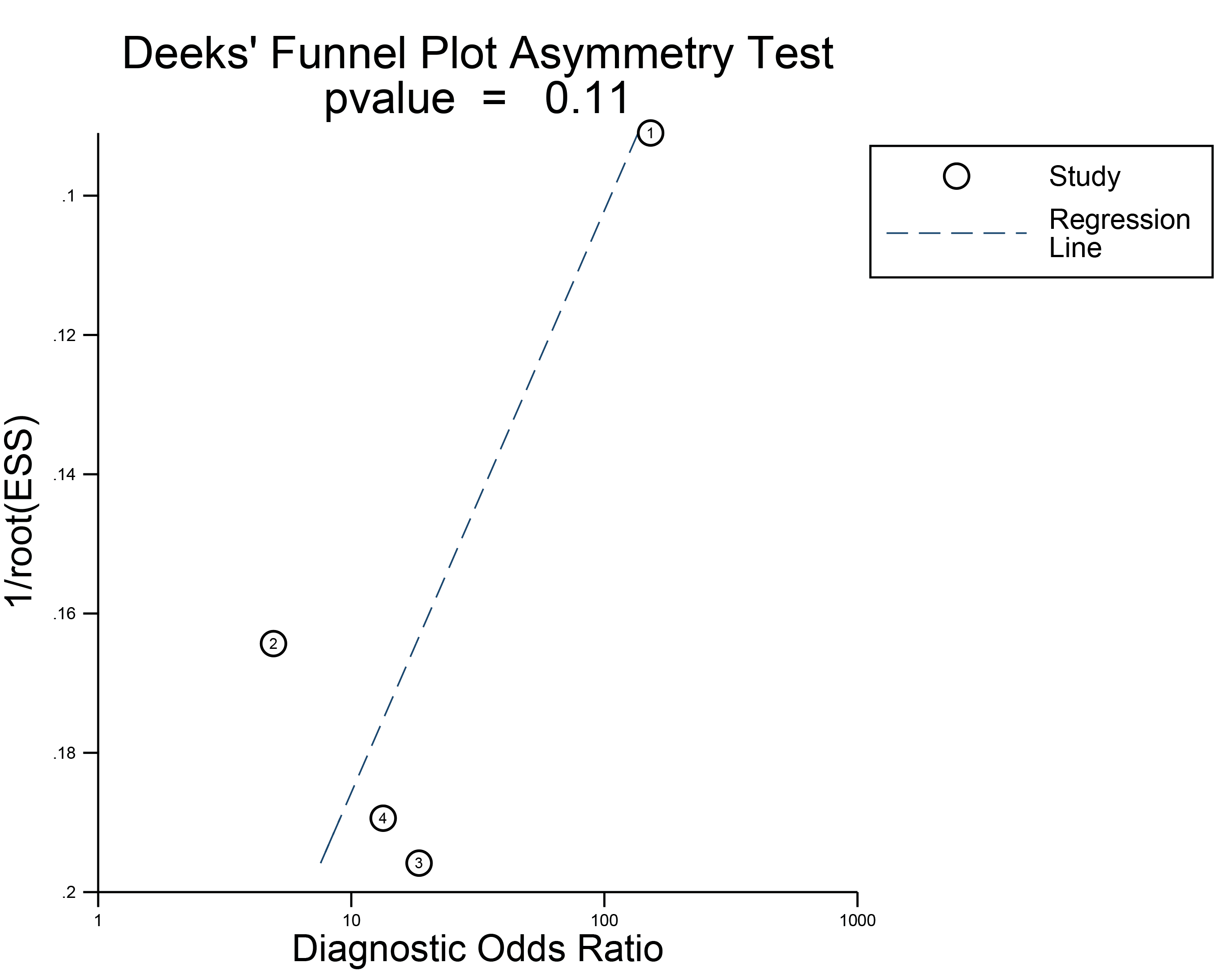

Supplement: S3 Fig — (TIF) [file pone.0125087.s003.tif]

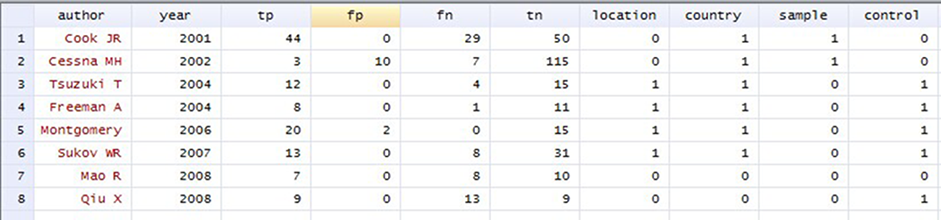

Supplement: S4 Fig — (TIF) [file pone.0125087.s004.tif]
